# En-Bloc Resection of Renal Cell Carcinoma With Tumor Thrombus Propagating Into the Intrapericardial Inferior Vena Cava: Efficacy and Safety of Transabdominal Approach

**DOI:** 10.7759/cureus.42394

**Published:** 2023-07-24

**Authors:** Theodoros Sidiropoulos, Stavros Parasyris, Vassiliki Ntella, Ioannis Margaris, Spyridon Christodoulou, Kassiani Theodoraki, Panteleimon Vassiliu, Vassilios Smyrniotis, Nikolaos Arkadopoulos

**Affiliations:** 1 4th Department of Surgery, Attikon University Hospital, National and Kapodistrian University of Athens, Athens, GRC; 2 1st Department of Anesthesiology, Aretaieion University Hospital, National and Kapodistrian University of Athens, Athens, GRC

**Keywords:** rcc with tumor thrombus in ivc, transabdominal approach, radical nephrectomy with ivc thrombectomy, renal cell carcinoma, neoplasmatic thrombus in intrapericardial ivc

## Abstract

Background: Renal cell carcinoma (RCC) is the most common primary kidney cancer. In up to 4-10% of patients, the tumor is complicated with a malignant thrombus extending to the inferior vena cava (IVC). Complete surgical excision of the RCC and the neoplastic thrombus can be curative. We aim to present a safe and feasible alternative transabdominal operative technique with the omission of thoracotomy, as applied in six patients diagnosed with RCC and IVC thrombus extending over the diaphragm.

Methods: This case series study was conducted in a tertiary university hospital in Athens, Greece. All six patients, who were operated on for RCC and a malignant thrombus exceeding in the intrapericardial IVC in our department from January 2009 until March 2020, were screened. Intraoperatively, the infrarenal and intrapericardial IVC were clamped simultaneously with the renal and liver blood inflow. Access to the intrapericardial IVC was obtained via the central tendon of the diaphragm. Intrathoracic extension of the tumor was confirmed by transesophageal or intraoperative ultrasonography. The intrathoracic IVC was exposed to direct vision and two finger palpation was applied to secure the clamping of the IVC above the tip of the thrombus. The tumor was resected through a longitudinal venotomy and the operation was completed on a standard radical nephrectomy.

Results: During the study period six patients presented with RCC and intrapericardial IVC thrombus. All patients, five female and one male, underwent radical nephrectomy combined with IVC thrombectomy, without the need for a thoracotomy. The mean age was 66 years old and the mean operative time was 122.5 minutes. Mean blood loss was 338 ml and only four of the patients were transfused with two units of RBC. Operative and hospital mortality was 0%. The hospital stay was seven (six to nine) days. Only one patient required readmission and reoperation 30 days later, due to intrapericardial herniation.

Conclusions: The proposed surgical technique may be curative in patients with advanced intracaval thrombus and helps reduce the associated morbidity, mortality, and the overall cost of more extended operations.

## Introduction

Renal cell carcinoma (RCC) is the most common primary malignancy that may be complicated with malignant thrombus formation propagating via the renal vein and inferior vena cava (IVC) upwards to intrathoracic structures [1]. Renal dissection en block with the tumor thrombus is mandatory, rendering the long-term survival rate for these patients similar to that of non-thrombotic patients [1,2].

The surgical intervention should be considered carefully, with regard to the extent of the thrombus. The cardinal maneuver to entrap the malignant projection inside the IVC can be easily accomplished as soon as it remains below the hepatorenal junction [3]. Infrarenal and retrohepatic clamping of the IVC alongside renal inflow control allows the resection of the tumor and its thrombus on a bloodless surgical field without serious hemodynamic changes by venotomy. However, when the thrombus enters the intrathoracic part of the IVC, defined as level IV, complete control of the IVC harboring the thrombus entails a transthoracic approach and vascular control of renal and liver blood inflow [4,5].

This surgical procedure may be associated with severe hemodynamic changes (acute drop of blood pressure) and thus, various techniques have been evaluated to optimize the return of blood from the lower parts of the body in order to maintain cardiac and cerebral blood perfusion [6]. Until recently, only a few studies in the literature addressed this complex surgical entity by controlling the intrathoracic and abdominal blood flow only through the abdomen [4-7].

Our study aims to show that transabdominal clamping of the intrapericardial IVC with a simultaneous Pringle maneuver can be used to safely and efficiently resect RCC with a level IV thrombus.

This article was previously posted as a preprint to the Research Square platform on the 19th of April 2022.

## Materials and methods

Patients

This case series study is a retrospective analysis of a prospectively maintained computerized database of patients who underwent radical nephrectomy with IVC thrombectomy at the surgical clinic of a tertiary academic center in Athens, Greece. Only patients who underwent surgery for RCC with level IV tumor thrombus from January 2009 to March 2020 in Attikon University Hospital were included. Patient data such as age, gender, location of the tumor, histology stage, operation time, blood loss, and transfusion were included. Details concerning the changes in mean arterial pressure (MAP), duration of vascular clamping, postoperative kidney function, management, and follow-up of these patients were also recorded.

Surgical technique

According to our proposed surgical technique, the abdomen was accessed with the patient in a supine position through a right or left subcostal or Makuuchi incision (if a sternotomy is anticipated). After a thorough assessment of the tumor resectability, the intrathoracic extension of the tumorous formation was confirmed by intraoperative or transesophageal ultrasonography, achieved by a 2-3 cm horizontal incision in the tendonous central portion of the diaphragm. The mobilization of the liver was routinely carried out for it to be easily rotated to both the left and right side and pulled down visualizing the hepatocaval junction. The lower half of the retrohepatic IVC was freed by dividing two of the three pairs of connecting veins to the liver from the IVC.

The tumor-bearing kidney was mobilized and prepared for radical nephrectomy securing inflow and outflow control. Vascular control of the infrarenal IVC with the aid of a vessel loop was completed first, even before the tumor mobilization from the surrounding tissues. When the primary tumor is located on the right kidney, occlusion of the left renal vein leaving free the arterial flow of the ipsilateral artery is not injurious to the renal parenchyma. By contrast, when the left kidney is to be resected, both the artery and the vein of the right kidney should be controlled, since in contrast to the left kidney with the existence of a collateral venous drainage system, the right kidney does not tolerate occlusion only of the outflow [5,6].

As soon as control of the infrarenal IVC and the renal veins was complete, the liver blood inflow was controlled by clamping the hepatoduodenal ligament and any accessory artery from the left gastric artery. The intrathoracic IVC was exposed to direct vision and two finger palpation was applied to secure the clamping of the IVC above the tip of the thrombus.

**Figure 1:**
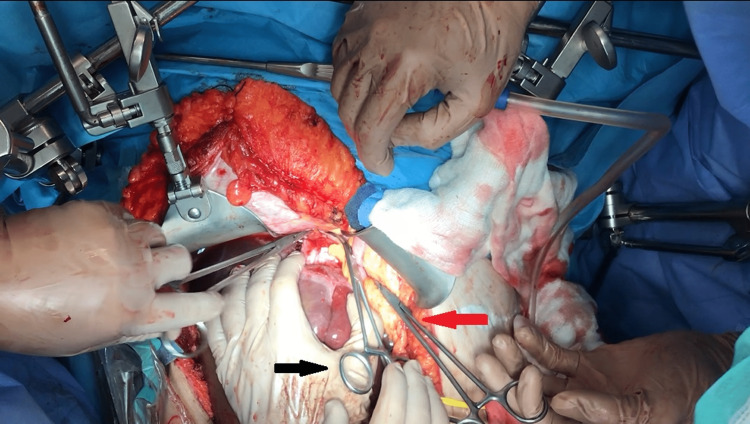
Intrapericardial clamping of the suprahepatic IVC (middle clamp: black arrow) in preparation for the removal of the tumor and IVC thrombus. The head of the patient is located on the left side of the picture. The left hand of the surgeon retracts the liver downwards. The inflow to the liver has also been occluded using the Pringle maneuver (top clamp: red arrow) in order to avoid liver congestion. IVC: inferior vena cava

Combined clamping of the intrathoracic IVC (Figure 1) and Pringle maneuver can lead to a significant drop in the MAP. In this case, partial clamping of the aorta at the level of the esophageal hiatus in coordination with the anesthetic team restored the hemodynamic disorder, securing mean arterial pressure MAP > 60mmHG.

**Figure 2:**
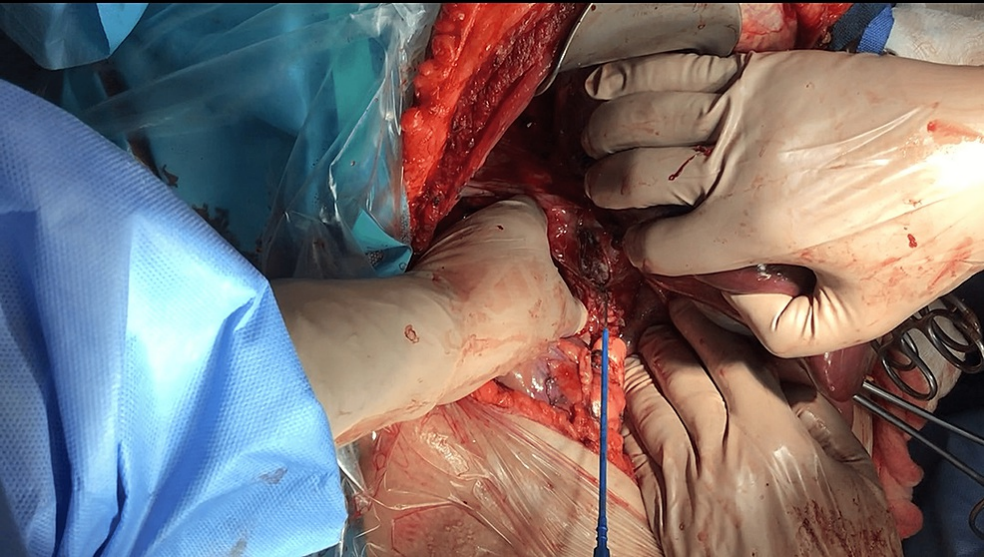
Opening of the thrombus - containing IVC using a 3-4 cm longitudinal incision above the level of the renal veins. IVC: inferior vena cava

We proceeded immediately with a 3-5 cm longitudinal venotomy (Figure 2) on the posterolateral aspect of the IVC incorporating the orifice of the renal vein hosting the thrombus. The floating thrombus was easily extracted from the IVC lumen, permitting us to clamp the retrohepatic IVC above the cavotomy and to unclamp swiftly the intrapericardial IVC and liver blood inflow. The tumor resection was completed on a standard radical nephrectomy.

The pericardial incision was left open, assuming that the left lobe of the liver would cover the defect of the diaphragm and protect it from post-operative herniation of abdominal viscera.

## Results

During the last eleven years, only six patients (one male and five females) with RCC and propagation of the malignant thrombus into the intrathoracic IVC received surgical treatment. The diagnosis was made using a computed tomography (CT) scan with intravenous (IV) contrast. A summary of the patient’s characteristics is presented in Table 1.

**Table 1:** Summary of patients’ characteristics RBC: red blood cells, MAP: mean arterial pressure, IVC: inferior vena cava

Patients’ characteristics	Patient 1	Patient 2	Patient 3	Patient 4	Patient 5	Patient 6
Age (years)	66	55	78	75	56	65
Sex	Female	Female	Female	Female	Female	Male
Histology stage	T3cN1M0	T4cN1M1	T3cN0M1	T3cN1M0	T3cN1M0	T3cN1M0
Location (right/left)	R	R	R	L	R	R
Level of thrombus	IV	IV	IV	IV	IV	IV
Operation time (min)	90	140	118	122	130	135
Blood losses (mL)	300	450	250	350	200	480
Transfusion RBC (units)	-	2	-	2	3	2
Complications	Pericardial Herniation	None	None	None	None	None
Discharge (day)	9^th^	8^th^	9^th^	8^th^	7^th^	6^th^
Changes of MAP during:						
Clamping of the infrarenal IVC plus renal vessels (mmHg)	105	85	90	100	85	75
Clamping of intrathoracic IVC plus Pringle (mmHg)	70	55	60	55	60	40
Duration of vascular clamping						
Intra-abdominal aorta (min)	-	4	-	5	-	7
Intrathoracic IVC plus Pringle (min)	5	4	5	4	6	5
Renal function (Creatinine 1^st^ day/6^th^ day post op) (mg/dL)	1.6/1.1	2.2/1.4	2.3/1.3	1.2/0.9	1.4/1.1	1.6/0.8

In five patients the tumor emanated from the right kidney and in one (No. 4) from the left. The mean age was 66 years old. In two of them the tip of the malignant formation was protruding 1 cm into the thoracic cavity and in four of them 2 cm, estimated by ultrasonography (US) and magnetic resonance angiography (MRA). In one patient (No. 3) a metastatic lesion 2 cm was confirmed in the left lung. In five patients the tumor was clear cell adenocarcinoma and in one (No. 2) it was transitional cell carcinoma (TCC). The malignant thrombus did not adhere to the IVC wall in all but one patient (No. 2), in whom the IVC was infiltrated close to the ipsilateral renal vein.

The mean operative time was 122.5 minutes. Mean blood loss was 338 mL and only four of the patients were transfused with two units of RBC. Intraoperatively, occlusion of the infrarenal IVC and renal vessels did not cause significant changes to the mean arterial pressure (10-20%). Combined clamping of the intrathoracic IVC and Pringle maneuver dropped the MAP 35-50%. Three patients (Nos. 2, 3, and 5) showed good response to the hemodynamic changes induced by the clamping of the infrarenal and intrapericardial IVC combined with renal and liver blood inflow. In three patients (Nos. 2, 4, and 6) the MAP dropped to 55, 55, and 40 mmHg respectively, which necessitated the partial clamping of the abdominal aorta at the esophageal hiatus for 4, 5, and 7 minutes respectively.

The clamping duration of the intra-abdominal IVC at various phases of the operation in patients Nos. 3, 4, 5, and 6 was 18, 17, 19, and 15 minutes respectively. In two cases (Nos. 1 and 2) the infrarenal IVC had to be obliterated. In one patient (No. 1) with benign thrombus of the infrarenal IVC and iliac vein, ligation was carried out. In patient No. 2, a 4 cm segment of the suprarenal IVC was resected, the left renal vein was ligated and the two IVC stumps sutured, leaving the defect unbridged.

**Figure 3:**
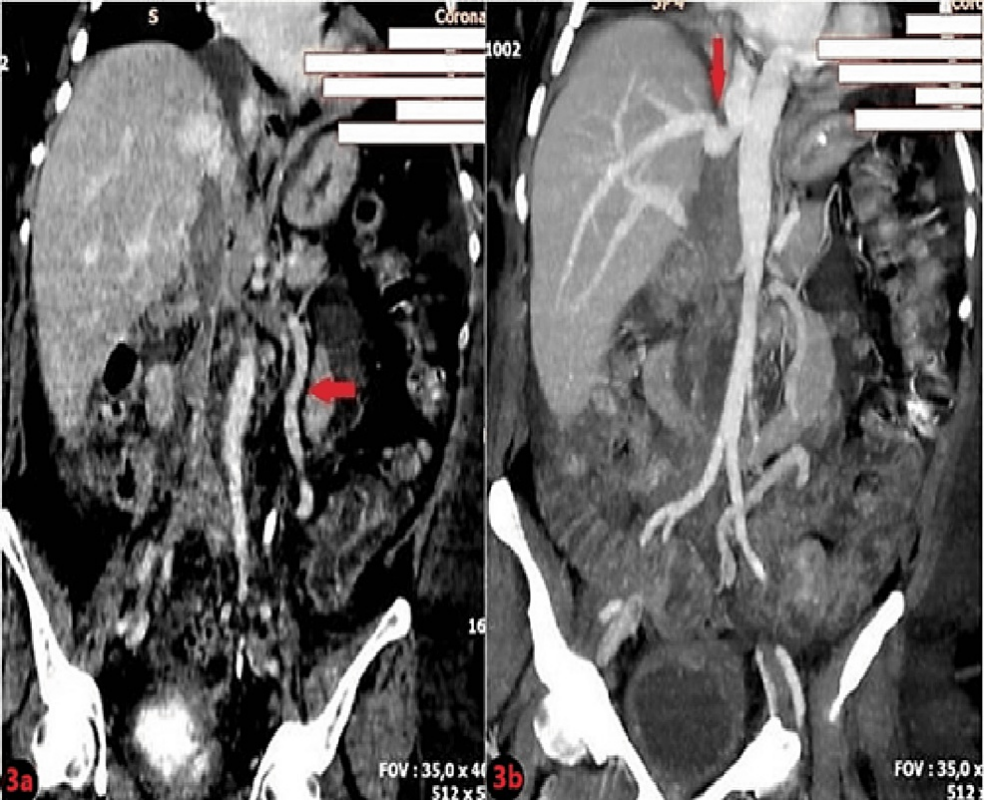
Postoperative abdominal and pelvic CT scan showing: a resection of the right kidney and tumor thrombus. The left renal vein was ligated and the spermatic vein (arrow) is left in place for the left kidney blood outflow. The inferior vena cava (IVC) is ligated below the level of the renal veins and the suprahepatic IVC is at the level of the junction with the hepatic veins (arrow). CT: computed tomography

The postoperative course was uneventful for all patients. None of the patients required postoperative stay at the Intensive Care Unit. A postoperative CT scan of the abdomen and pelvis of the patient (No. 1) on the fifth postoperative day is shown in Figure 3.

The hospital stay was seven (six to nine) days. Only patient (No. 1) required readmission and reoperation 30 days later, due to intrapericardial herniation. Nineteen months after the procedure, the patient is doing well.

All patients were compliant with their follow-up appointment and imaging. The mean follow-up was 19 months. None of the patients received adjuvant chemotherapy. All patients remain functionally well and independent and none has had a local or systemic recurrence of the disease.

## Discussion

The technique presented in this setting of patients demonstrates that the intrathoracic IVC approach via a small incision at the diaphragm provides an excellent view of the intrapericardial IVC and its content, allowing the surgeon to put a clamp above the tip of the thrombus and avoid a thoracotomy, sternotomy or a thoracoabdominal procedure. The mean operative time is 122.5 minutes which is acceptable and significantly lower than it could be for a patient to undergo cardiopulmonary bypass (CBP) in our setting.

The hemodynamic alterations with blood pressure < 60 mmHg were effectively reversed with anesthetic intervention and partial aortic occlusion in three of the patients for 4, 5, and 7 minutes. The malignant thrombus was swiftly extracted and the intrapericardial clamp was replaced by clamping the retrohepatic IVC and restoring the liver blood inflow in less than 6 minutes. The technique is based on experience in advanced liver transplant and was developed in order to achieve lower morbidity and mortality than expected with thoracotomy.

In 4-10% of the patients with RCC, projection of the tumor as a malignant thrombus via the renal vein may be encountered as high as the thoracic cavity [8]. Radical renal resection with extraction of the intraluminal malignancy should be pursued since the five-year survival is comparable to that of counterparts without malignant thrombus [2,3,9]. From the surgical point of view, the extent of the malignant thrombus will define the surgical intervention. There is a variety of classification systems used for venous tumor thrombus in RCC patients. The most widely accepted system in the literature describing the propagation of the thrombotic process is the Mayo staging system (levels 0 to IV) [10]. Based on this system, all six of our patients had a level IV tumor thrombus, extending beyond the diaphragm.

Extraction of the malignant thrombus when it remains below the hepatocaval junction (levels 0, I, and II) requires complete vascular control at the intra-abdominal IVC by clamping the infrarenal and retrohepatic IVC, concomitantly with the renal vessels [11]. The entrapped thrombus can be easily extracted in a bloodless surgical field via a 3-5 cm longitudinal incision at the IVC tailored to incorporate a rim of the renal vein harboring the thrombus, so as to avoid IVC stenosis [6,12,13].

However, in 1% of the patients with RCC, the malignant thrombus propagates above the hepatocaval junction into the thoracic cavity (level IV) [14]. Under this circumstance, complete vascular control of the IVC harboring the thrombus necessitates clamping of the intrapericardial IVC alongside infrarenal IVC, and renal and liver blood inflow [4]. Occlusion of the intrathoracic IVC combined with the Pringle maneuver may be implicated with hemodynamic instability and, in 20% of cases, is barely tolerated for a short period of time [6,15]. Therefore, veno-venous bypass from the femoral vein to the auxiliary or jugular vein is widely used as the simplest way to enhance the return of blood volume from the lower body to the heart and brain [15-19].

Cardiac arrest under hypothermia, deep hypothermic circulatory arrest (DHCA) has been used to protect the liver and kidney from warm ischemic injuries in more complex situations. Chen et al. show in their study that patients with level III or IV IVC thrombus RCC can be treated with radical nephrectomy and thrombectomy using CBP combined with DHCA [15]. Even though recent advances in the field of cardiothoracic surgery and the utilization of CPB machines have helped decrease postoperative complications, perioperative morbidity remains high [19,20]. Moreover, these procedures are time-consuming and the need for a multidisciplinary team cannot always be met.

Similar to our purpose and in an effort to overcome the above-stated obstacles and decrease postoperative morbidity and mortality rates, a few different surgical maneuvers have been proposed. The University of Miami technique described by Ciancio et al. is a transabdominal approach using specialized liver transplantation techniques. With dissection of the diaphragm, the intrapericardial IVC is revealed. Under the guidance of transesophageal echocardiography (TEE), the tumor thrombus, either adherent or mobile, is removed without the use of CPB [21].

In the study from Fukazawa et al., only five patients out of a total of 70 (7.1%) required CPB (two with level III and three with level IV), showing that the avoidance of extracorporeal circulation is possible with the cooperation of surgeons and anesthesiologists and good utilization of the TEE [5]. This technique comes with minimal complications and is suitable for patients who cannot tolerate the CPB approach, proving that a transabdominal approach as presented is feasible.

Our technique has the advantage that it can be executed transabdominally and should be considered for patients with floating malignant nonadherent thrombus. The infrarenal control of the IVC did not alert us. Vein flow collateralization compensated the occlusion of infrarenal IVC. The cardinal maneuver of simultaneous occlusion of the suprahepatic IVC and hepatic blood inflow can cause hemodynamic changes that require a prompt anesthetic response and partial clamping of the abdominal aorta for a short period to keep MAP > 60 mmHg and secure cardiac and cerebral blood perfusion without the use of excessive IV crystalloids and vasopressors [22].

The limitations of the study are the retrospective character and the small portion of patients in a large period. Although there are certain limitations, it is a safe technique that could be adopted by experienced surgeons in settings where there is time limitation in the operation room and a lack of a thoracic surgical team.

## Conclusions

RCC with a tumor thrombus extending into the IVC is a rare surgical entity. The transabdominal approach seems to be feasible for complete tumor and thrombus resection. Nonetheless, a multidisciplinary approach is required, and the surgical team should be experienced in liver surgery or liver transplantation techniques.
